# Association between volatile organic compound co-exposure and the prevalence of rheumatoid arthritis: a nationwide cross-sectional study

**DOI:** 10.3389/fpubh.2025.1694503

**Published:** 2025-11-10

**Authors:** Tian Ren, Erye Zhou, Tao Cheng, Mingjun Wang, Yufeng Yin, Jian Wu, Weichang Chen

**Affiliations:** 1 Department of Rheumatology and Immunology, The First Affiliated Hospital of Soochow University, Suzhou, Jiangsu, China; 2 Department of Gastroenterology, The First Affiliated Hospital of Soochow University, Suzhou, Jiangsu, China

**Keywords:** volatile organic compounds, VOCs, mVOCs, rheumatoid arthritis (RA), lymphocyte-to-monocyte ratio (LMR)

## Abstract

**Background:**

Environmental contaminants, especially volatile organic compounds (VOCs) and their metabolites (mVOCs), are of significant interest for treating autoimmune diseases due to their potential immunomodulatory effects. This study aimed to assess the association between urinary mVOCs and the risk of rheumatoid arthritis (RA) in U.S. adults.

**Methods:**

A total of 4,622 adults, including 296 participants with RA, were included in the present study utilizing data from the National Health and Nutrition Examination Survey (NHANES) from 2005 to 2020. Sixteen mVOCs were selected for the analysis while controlling for potential confounders. Weighted logistic regression models were employed to assess the association between individual mVOCs and RA risk. Least absolute shrinkage and selection operator (LASSO) regression was used to select mVOCs and covariates most pertinent to the prevalence of RA for further analyses. Then, weighted quantile sum (WQS) regression and quantile g-computation (qgcomp) models were used to estimate associations between the mVOC mixture and RA. Mediation analyses were performed to examine the effect of inflammatory indices on these relationships.

**Results:**

In single-pollutant models, levels of most mVOCs were greater in the RA patients than in the patients without arthritis. Furthermore, multi-pollutant models unveiled a positive effect of the mVOC mixture on the risk of RA in both WQS regression (OR: 1.37; 95% CI: 1.12, 1.68; *P* = 0.002) and qgcomp (OR: 1.23; 95% CI: 1.07, 1.49; *P* = 0.034) models. This effect was notably stronger for female participants. The lymphocyte-to-monocyte ratio (LMR), a surrogate for inflammatory markers, mediated the association between the mVOC mixture and the prevalence of RA with a mediated proportion of 4.65%.

**Conclusions:**

This study supports the substantial connection between VOC co-exposure and the risk of RA, with inflammation potentially acting as a mediator in this relationship.

## 1 Introduction

Rheumatoid arthritis (RA), is one of the most prevalent systemic autoimmune diseases, is characterized by chronic joint inflammation, leading to progressive joint damage and disability, with increased mortality (1). RA has a global prevalence of approximately 0.5% among adults, and women are 2 to 3 times more likely than men to develop this disease (2). Although it can manifest at any age, the most common age range for RA onset is between 50 and 59 years old (2). The disease burden of RA extends beyond joint disorders, with patients exhibiting an increased risk for cardiovascular disease, infections, and psychological disorders, underscoring its significant impact on public health and healthcare systems worldwide (2).

The pathogenesis of RA is not fully understood; however, it is widely accepted as a multifactorial disease in which genetic susceptibility interacts with environmental triggers (3). Although genetic factors contribute substantially to the risk of RA, they do not fully account for the disease occurrence, which implies a critical role for environmental factors (3). Among these, smoking is the most well-documented risk factor (4). Other environmental exposures, such as air pollution, occupational hazards, and microbial agents, have also been associated with the onset and progression of RA (5, 6).

Volatile organic compounds (VOCs) are a diverse group of carbon-based chemicals that readily evaporate at ambient temperature (7). Human exposure to VOCs can occur through various routes, including inhalation, ingestion, and dermal absorption, originating from a wide array of sources, such as industrial emissions, vehicular exhaust, building materials, and use of consumer products (8). Once in the body, VOCs can be metabolized into a range of metabolites (mVOCs), which might exert toxic effects and have been implicated in the pathogenesis of several diseases, especially systemic autoimmune diseases (7–9) and joint disorders (10).

Considering the ubiquitous nature of VOCs and their potential immunomodulatory effects, recent studies have elucidated the intricate relationship between RA and exposure to VOCs; however, these relationships present a complex picture. Research by Lei et al. suggests that certain single mVOCs, such as AMCA and HPMA, may be involved in the pathogenesis of RA (11). On the other hand, a concurrent study by Beidelschies et al. highlighted that environmental toxicants, including polycyclic aromatic hydrocarbons (PAHs) but not VOCs, are associated with an increased risk of RA (12).

While these works have significantly contributed to our understanding of the potential impact of mVOCs on RA, several critical questions remain to be explored to fully clarify the role of these compounds in RA. For instance, as with most environmental exposures, VOC exposures frequently encompass a mixture of different VOCs that can interact with one another, potentially leading to synergistic or interactive impacts on health outcomes (13). Therefore, it is vital to assess the combined effects of these co-exposures to better understand their health impacts. Moreover, delineating the mediating factors, particularly the role of inflammatory processes, is important for obtaining a comprehensive understanding of how environmental exposures contribute to the emergence and progression of RA.

Therefore, our research aims to bridge current knowledge gaps by analyzing the cumulative impact of mVOC mixtures on RA prevalence in a representative U.S. adult cohort. Moreover, we aimed to analyze whether inflammatory markers act as mediators of the correlation between the mVOC and increased risk of RA.

## 2 Methods

### 2.1 Study design

The data used were obtained from NHANES, a program committed to assessing the health and nutritional status of the civilian population in the U.S. This program employs a complex, multistage sampling method and has been gathering extensive data from a nationally representative sample biannually since 1999, covering various counties through both mobile examination centers (MECs) and in-home interviews. The Centers for Disease Control and Prevention (CDC) provides comprehensive information on the NHANES methodology, design, and participant recruitment. This investigation was conducted in adherence to the STROBE guidelines tailored for cross-sectional studies and received approval from the Research Ethics Review Board at the National Center for Health Statistics (NCHS). Informed consent was obtained from all individual participants involved in the study.

### 2.2 Study population

The study consolidated data across five NHANES cycles (2005–2006, 2011–2012, 2013–2014, 2015–2016, and 2017–2020), amounting to a total of 55,810 participants. Individuals younger than 20 years were excluded. Those with missing mVOC values or apparent outliers (exceeding the 99th percentile) were also excluded from the analysis to minimize potential bias arising from extreme data. Additional exclusions were made for missing data on covariates, complete blood count and RA status. Following these criteria for inclusion and exclusion, the final analytical sample included 4,622 participants (Supplementary Figure S1).

### 2.3 Assessment of RA

RA status was assessed using a disease questionnaire administered prior to the physical examination. This involved a computer-assisted face-to-face interview asking participants aged >20 years the following question: “Has a doctor or other health professional ever told you that you had arthritis?”. Those who answered affirmatively were then asked “Which type of arthritis was it?”, and persons who responded with RA were classified as having RA. Participants with incomplete data regarding arthritis and RA status, as well as those with missing information on relevant covariates, were excluded from the analyses.

### 2.4 Measurements of mVOCs

Urine specimens were obtained from the participants, who were not required to adhere to any fasting or dietary restrictions. Each specimen was deposited into either polystyrene cryovial tubes or polypropylene centrifuge tubes. A minimum volume of 0.25–0.5 mL was obtained, with an assay-specific aliquot of 50 μL. After collection, the samples were promptly chilled and transferred to a storage facility where they were maintained at −20 °C and at −70 °C until analysis. Urinary mVOCs were quantified using an advanced ultra-performance liquid chromatography (UPLC) system paired with electrospray ionization tandem mass spectrometry (ESI-MS/MS) (14).

Data are reported in concentration units (ng/mL) and were normalized to creatinine levels (μg/g creatinine) to account for urine dilution variability among specimens. A comprehensive outline of the analytical procedures utilized in the laboratory is accessible at the CDC website via NHANES laboratory methods. For instances in which the analyte concentrations fell below the established lower limit of detection (LLOD), the reported values were assigned as the LLOD divided by the square root of two (LLOD/√2) (15). In NHANES, a total of 29 different urinary mVOCs are analyzed. However, we omitted 13 mVOCs from our analysis due to their low detection rate (50% or less) to ensure the representativeness of the data and the reliability of the findings. Ultimately, 16 urinary mVOCs were considered for analysis (Supplementary Tables S1, S2).

### 2.5 Covariates and inflammatory markers

Covariates were selected due to their established association with arthritis, as indicated by previous studies (10, 11, 16). Selection of covariates included demographic details such as age, sex (male and female), racial/ethnic background (Mexican American, other Hispanic, Non-Hispanic White, Non-Hispanic Black, and other/multiracial), educational attainment (ranging from less than 9th grade to college graduate or higher), marital status (categorized as married/living with partner, widowed/divorced/separated, or never married), family income in relation to the poverty level as indicated by the poverty income ratio (PIR) (0–1.29, 1.3–3.49, and ≥3.5), and status of health insurance coverage (either insured or uninsured). Clinical parameters included body mass index (BMI), which was defined as underweight (<18.5 kg/m^2^), normal weight (18.5–24.9 kg/m^2^), overweight (25–29.9 kg/m^2^), and obese (≥30 kg/m^2^), along with waist circumference (cm). Comorbid conditions such as hypertension and diabetes were identified through participants' self-reported diagnoses that had been confirmed by a physician. The inflammatory marker utilized in the study was the lymphocyte-to-monocyte ratio (LMR), which was calculated by dividing the number of lymphocytes in the peripheral blood by the number of monocytes.

### 2.6 Statistical analyses

Baseline demographic characteristics were compiled and summarized for the overall population, and comparisons were drawn between groups based on the presence of RA or absence of arthritis (RA vs. non-arthritis). Continuous variables are presented as weighted means accompanied by standard deviations (SDs) or, alternatively, medians paired with interquartile ranges (IQRs). To assess differences between groups, independent *t*-tests and Wilcoxon rank-sum tests were employed as appropriate for the data distribution. Categorical variables were quantified as counts (percentages), and chi-square tests were used to determine the significance of differences observed between groups. All 16 mVOCs were subjected to natural logarithm (ln) transformation to approximate a normal distribution. Following this transformation, the data were standardized and divided into four quartiles (Q1, Q2, Q3, and Q4) to facilitate subsequent analyses.

In single-pollutant models, weighted multivariate binary logistic analyses were conducted to examine relationships between individual mVOCs and the risk of RA, and odds ratios (ORs) and 95% confidence intervals (CIs) are reported. Restricted cubic spline (RCS) analyses (with 3 automatically selected knots) were also conducted to investigate non-linear relationships. Pearson correlation tests were used to examine interrelationships between mVOCs by assessing the strength and direction of their mutual associations.

Given the significant correlations and multicollinearity observed among the 16 mVOCs, which can produce unstable estimates and reduce the interpretability of traditional regression models, we employed the least absolute shrinkage and selection operator (LASSO) regression (17). This machine learning method is particularly advantageous for our analysis as it performs both variable selection and regularization simultaneously. By applying a penalty function, LASSO shrinks the coefficients of less influential predictors toward zero, allowing us to identify a more robust and parsimonious subset of mVOCs and covariates for the subsequent mixture effect analyses with WQS and qgcomp.

To assess the joint effect of the chemical mixture on RA prevalence, the variables selected by LASSO were incorporated into a weighted quantile sum (WQS) regression analysis. We chose this approach to model a more realistic environmental exposure scenario, as humans are typically exposed to multiple chemicals simultaneously rather than in isolation. The WQS model collapses the high-dimensional set of correlated mVOCs into a single, empirically weighted index. A key advantage of this method is that it not only estimates the overall effect of the mixture but also identifies the individual components that contribute most significantly to this association by examining their respective weights.

To address constraints associated with the WQS regression approach, particularly regarding the directionality of associations, we applied the quantile g-computation (qgcomp) model. The adopted methodology merges the inferential framework of WQS regression with the flexible characteristics of g-computation, effectively sidestepping the constraints imposed by assumptions of directional homogeneity. Notably, it separates the adjustment for confounders from the estimation of effects, which can enhance the clarity of the analysis. Moreover, it allows for a causal inference perspective on the parameters estimated (18, 19). Causal mediation analysis was performed to ascertain whether inflammatory indices act as mediators of the relationship between the mVOC and RA risk, including the extent of such mediation.

Statistical estimates were calibrated to accommodate the complex sampling design of NHANES, utilizing the specific sample weights and stratification information that accompany the survey data. However, these same adjustments were not incorporated into the WQS regression or the qgcomp model due to their incompatibility with complex survey designs. Statistical analyses were performed with R software, version 4.3.2 (R Foundation for Statistical Computing, Vienna, Austria), and a two-sided *P*-value of less than 0.05 was considered to indicate statistical significance.

## 3 Results

### 3.1 Demographic characteristics and mVOC levels of the study participants

The study analyzed the baseline characteristics of 4,622 participants, including 2,303 females (48.77%) and 2,319 males (51.23%), with an average age of 44.00 years. In the RA group (*n* = 296), the average age was slightly older than that in the control group, with a considerable percentage of participants being in the ≥60 years age group. The racial distribution of the RA group revealed a greater percentage of Non-Hispanic White individuals. The mean BMI for the RA group was 30.86 kg/m^2^, indicating a greater prevalence of overweight and obesity within this subgroup. The RA group also showed different patterns of educational attainment and health insurance coverage than did the non-arthritic group. Moreover, the poverty income ratio was lower in the RA individuals, indicating a potential socioeconomic impact on RA prevalence. Smoking and alcohol consumption rates differed slightly between the groups, with a higher percentage of non-smokers and non-excessive drinkers in the RA group (Table 1). Supplementary Figure S2 shows the histogram of the mVOC distribution across the NHANES cycles. Supplementary Table S3 summarizes the percentile distributions and missing values (percentages) of the 16 studied mVOCs in these cycles.

**Table 1:** Baseline characteristics of study participants.

**Characteristic**	**Overall (*n* = 4,622)**	**Non-arthritis (*n* = 4,326)**	**RA (*n* = 296)**	***P*-value**
**Sex**				
Female	2,303 (48.77%)	2,145 (48.47%)	158 (54.79%)	0.333
Male	2,319 (51.23%)	2,181 (51.53%)	138 (45.21%)	
Age (years)	44.00 (15.80)	43.34 (15.62)	57.34 (13.35)	<0.001
**Age group**				
20–60 years	3,487 (81.90%)	3,365 (83.38%)	122 (52.08%)	<0.001
≥60 years	1,135 (18.10%)	961 (16.62%)	174 (47.92%)	
**Race/ethnicity**				
Mexican American	728 (8.52%)	688 (8.57%)	40 (7.63%)	0.044
Other Hispanic	403 (6.46%)	381 (6.53%)	22 (5.12%)	
Non-Hispanic White	1,800 (66.01%)	1,680 (65.99%)	120 (66.40%)	
Non-Hispanic Black	1,072 (11.18%)	977 (10.94%)	95 (16.11%)	
Other/multiracial	619 (7.83%)	600 (7.98%)	19 (4.73%)	
BMI (kg/m^2^)	28.74 (6.45)	28.63 (6.39)	30.86 (7.24)	0.001
**BMI group**				
Underweight	64 (1.05%)	61 (1.07%)	3 (0.65%)	0.013
Normal	1,319 (29.72%)	1,269 (30.35%)	50 (16.99%)	
Overweight	1,552 (33.69%)	1,448 (33.51%)	104 (37.42%)	
Obese	1,687 (35.54%)	1,548 (35.07%)	139 (44.95%)	
Waist circumference (cm)	98.70 (15.98)	98.36 (15.90)	105.47 (16.17)	<0.001
**Educational attainment**				
Less than 9th grade	354 (3.69%)	317 (3.54%)	37 (6.65%)	0.010
9–11th grade	526 (7.78%)	486 (7.72%)	40 (8.99%)	
High school graduate	1,002 (21.36%)	932 (21.33%)	70 (21.95%)	
Some college	1,413 (30.56%)	1,305 (30.03%)	108 (41.24%)	
College graduate or above	1,327 (36.61%)	1,286 (37.37%)	41 (21.17%)	
**Health insurance**				
Insured	3,611 (81.48%)	3,345 (81.05%)	266 (90.16%)	<0.001
Uninsured	1,011 (18.52%)	981 (18.95%)	30 (9.84%)	
PIR	3.10 (1.64)	3.12 (1.64)	2.63 (1.66)	0.012
**PIR group**				
0–1.29	1,281 (19.48%)	1,166 (18.91%)	115 (31.04%)	0.026
1.3–3.49	1,740 (34.69%)	1,643 (34.82%)	97 (32.14%)	
≥3.5	1,601 (45.82%)	1,517 (46.27%)	84 (36.82%)	
**Smoking status**				
Non-smoker	2,765 (61%)	2,626 (61.50%)	139 (50.98%)	0.053
Smoker	1,857 (39%)	1,700 (38.50%)	157 (49.02%)	
**Alcohol consumption**				
Excessive	752 (21.46%)	711 (21.51%)	41 (20.52%)	0.743
Non-excessive	3,870 (78.54%)	3,615 (78.49%)	255 (79.48%)	
**Hypertension**				
Hypertension	1,344 (25.16%)	1,168 (24.04%)	176 (47.77%)	<0.001
Non-hypertension	3,278 (74.84%)	3,158 (75.96%)	120 (52.23%)	
**Diabetes**				
Diabetes	495 (8.36%)	418 (7.63%)	77 (23.03%)	<0.001
Non-diabetes	4,127 (91.64%)	3,908 (92.37%)	219 (76.97%)	

Continuous variables are expressed as the mean ± standard deviation (SD), while categorical variables are presented as numbers (%). All estimates were adjusted for sample weights and complex survey designs, with means and percentages further adjusted for the survey weights of NHANES. RA, rheumatoid arthritis; BMI, body mass index; PIR, poverty income ratio.

### 3.2 Associations of individual mVOCs with RA

Table 2 shows the concentrations of 16 different mVOCs stratified by the presence of RA. Participants diagnosed with RA exhibited elevated concentrations of the majority of the mVOCs assessed, except for BMA, BPMA, 2MHA and 34MH. The ln-transformed values of the mVOCs according to the status of RA are displayed in Supplementary Table S4. The association between single mVOCs and the prevalence of RA was assessed through weighted logistic regression analysis adjusted for covariates and is presented in Supplementary Table S5. According to the different models, certain mVOCs were significantly associated with the prevalence of RA. For instance, when considering the ln-transformed variable, AAMA showed an increasing trend in ORs across quartiles, with a significant trend in all three models (model 1: *P* for trend = 0.037; model 2: *P* for trend = 0.003; model 3: *P* for trend = 0.006). The continuous form of AAMA was also associated with an increased prevalence of RA (all *p* < 0.05 in the three models), and a similar trend was found between the prevalence of RA and other mVOCs, including AMCA, CEMA, CYMA, DHBM, HPMA, MHB3, PHGA, and HPMM (all *P*-values for trend <0.05).

**Table 2:** Concentrations of mVOCs according to RA status.

**mVOCs (urine, ng/mL)**	**Overall (*n* = 4,622)**	**Non-arthritis (*n* = 4,326)**	**RA (*n* = 296)**	***P*-value**
AAMA	45.93 (24.40, 88.92)	45.60 (24.20, 87.54)	55.64 (31.74, 104.35)	0.003
AMCA	135.00 (66.70, 279.00)	132.00 (66.00, 270.00)	233.05 (99.93, 422.26)	<0.001
ATCA	93.91 (42.30, 187.00)	91.65 (42.00, 186.00)	128.00 (55.32, 220.46)	0.014
BMA	5.97 (3.05, 10.70)	5.91 (3.00, 10.70)	6.40 (3.69, 9.89)	0.295
BPMA	3.42 (0.85, 9.27)	3.43 (0.85, 9.33)	2.73 (0.85, 7.93)	0.224
CEMA	85.78 (42.70, 156.00)	83.50 (42.14, 154.00)	126.00 (79.09, 213.00)	<0.001
CYMA	1.51 (0.74, 4.19)	1.49 (0.73, 4.04)	1.71 (0.98, 28.80)	0.008
DHBM	280.00 (152.00, 458.28)	276.00 (151.00, 455.00)	360.67 (208.72, 504.11)	<0.001
HPMA	214.00 (107.00, 413.00)	211.00 (106.00, 410.00)	256.51 (164.26, 472.36)	0.002
HPM2	28.00 (14.50, 52.59)	27.40 (14.40, 52.10)	31.93 (18.11, 61.73)	0.025
MADA	125.00 (68.40, 216.00)	125.00 (68.10, 215.00)	137.75 (82.47, 238.44)	0.023
2MHA	27.50 (12.80, 66.30)	27.50 (12.80, 66.10)	32.20 (12.51, 71.78)	0.622
34MH	178.00 (76.97, 445.00)	178.00 (75.88, 443.00)	181.34 (90.95, 544.60)	0.365
MHB3	4.41 (2.30, 9.21)	4.32 (2.28, 8.95)	6.15 (3.25, 13.70)	0.001
PHGA	183.00 (95.44, 317.00)	180.00 (93.74, 316.00)	221.00 (137.00, 328.33)	<0.001
HPMM	205.06 (109.00, 382.00)	203.00 (107.00, 375.98)	241.05 (169.93, 571.00)	<0.001

All values are presented as medians (IQRs). mVOCs, metabolites of volatile organic compounds; RA, rheumatoid arthritis.

**Figure 1:**
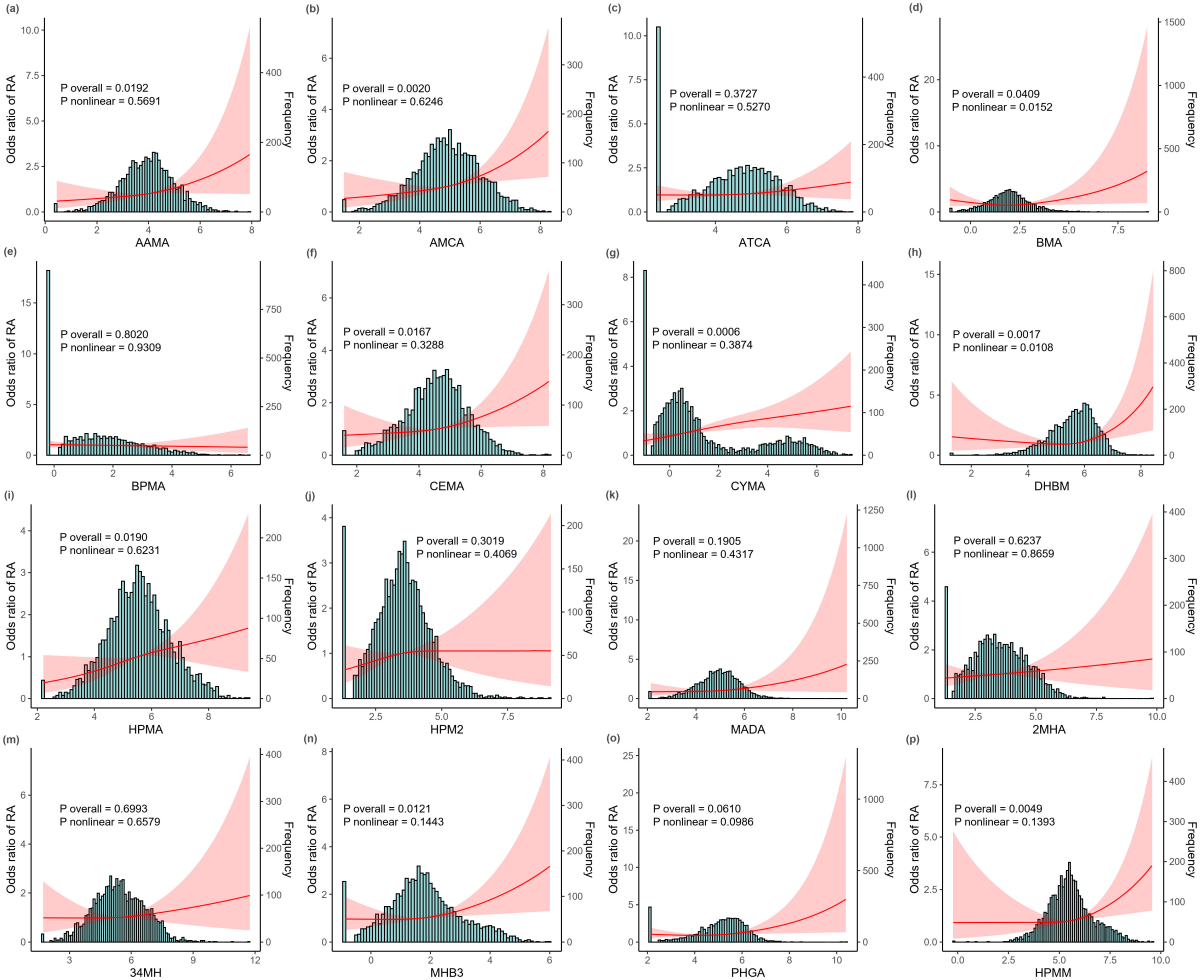
The dose—response relationship between ln-transformed metabolites of volatile organic compounds (mVOCs) and the risk of rheumatoid arthritis (RA) detected using restricted cubic spline (RCS) models. **(a)** AAMA, **(b)** AMCA, **(c)** ATCA, **(d)** BMA, **(e)** BPMA, **(f)** CEMA, **(g)** CYMA, **(h)** DHBM, **(i)** HPMA, **(j)** HPM2, **(k)** MADA, **(l)** 2MHA, **(m)** 34MH, **(n)** MHB3, **(o)** PHGA, and **(p)** HPMM. All analyses were adjusted for age, sex, race, body mass index (BMI), waist circumference, education, health insurance, marital status, poverty income ratio (PIR), smoking status, alcohol intake, hypertension, and diabetes status. The red line illustrates the odds ratio (OR) of RA, while the red shaded region denotes 95% confidence intervals (CIs). mVOCs, metabolites of volatile organic compounds; RCS, restricted cubic spline; OR, odds ratio; CI, confidence interval; BMI, body mass index; PIR, poverty income ratio.

Figure 1 illustrates the generalized linear regression analysis using RCS analyses to explore the dose–response relationship between urinary mVOCs and the prevalence of RA. The RCS models revealed specific mVOCs (such as AAMA, AMCA, BMA, CEMA, CYMA, DHBM, HPMA, MHB3, and HPMM) to be significantly associated with RA (P overall <0.05). Notably, several mVOCs, including BMA and DHBM, exhibited a non-linear relationship with RA (*P* non-linearly <0.05).

### 3.3 Correlations among individual mVOCs

**Figure 2:**
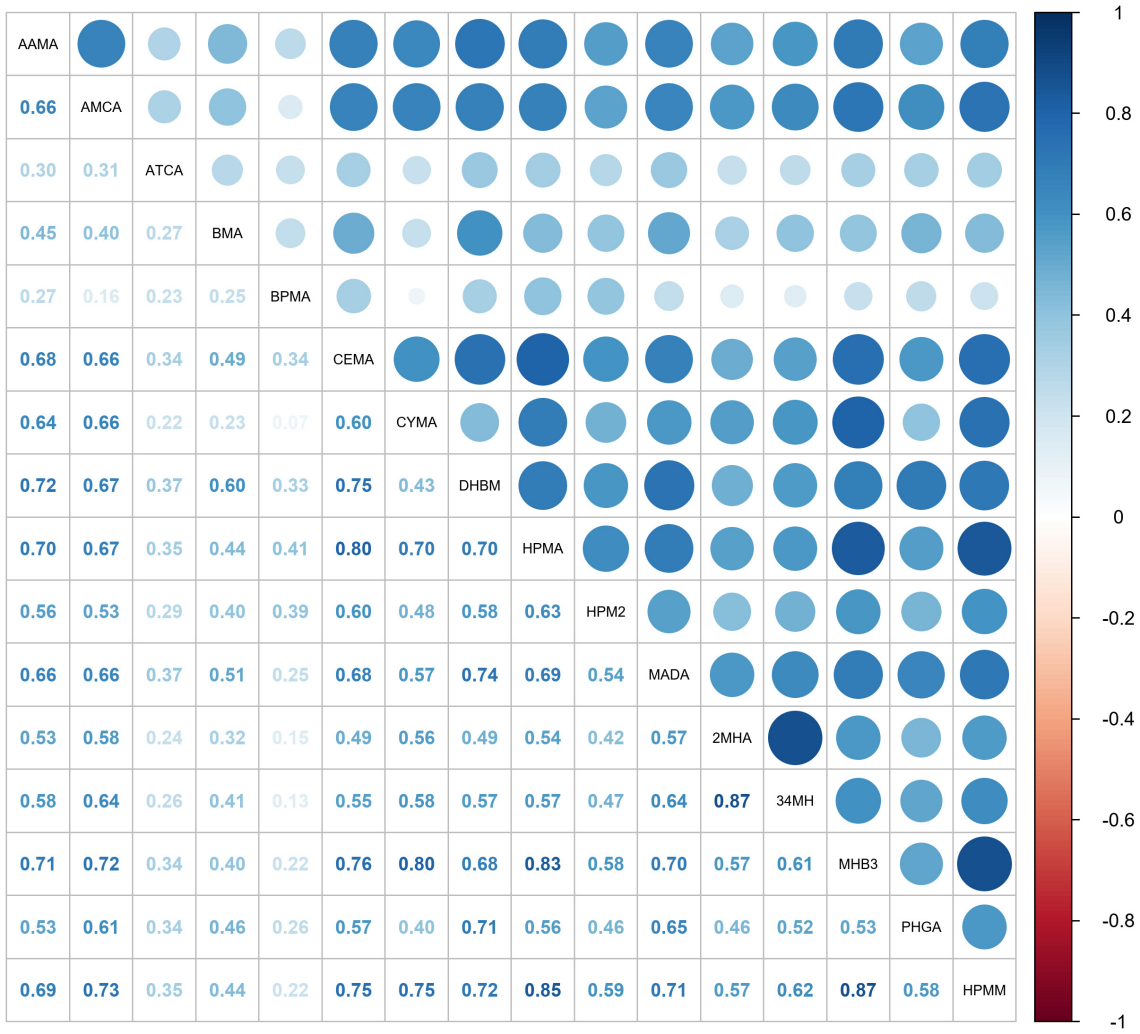
The matrix displays Spearman correlation coefficients (ρ) for the ln-transformed concentrations of 16 mVOCs. Darker shades reflect stronger correlations, with blue indicating positive relationships and red negative ones. mVOCs, metabolites of volatile organic compounds.

Spearman correlation analysis was also conducted to evaluate relationships between urinary mVOCs. Figure 2 presents a correlation matrix that highlights several mVOCs with significant positive or negative correlations. A strong correlation was identified between mVOCs derived from identical parent compounds: 2MHA and 34MHA (metabolites of xylene), with a correlation coefficient (*r*) of 0.87, and between CEMA and HPMA (metabolites of acrolein) (*r* = 0.80). In addition, our analysis revealed notable correlations between CYMA and MHB3 (*r* = 0.80), between HMPA and MHB3 (*r* = 0.83), between HPMA and HPMM (*r* = 0.85), and between MHB3 and HPMM (*r* = 0.87). These strong correlations suggest the presence of multicollinearity among these mVOCs.

### 3.4 Identification of mVOCs and covariates more relevant to RA

LASSO regression, in which penalty functions are applied to shrink less relevant mVOC coefficients toward zero, was used to effectively select those with more substantial associations with the risk of RA. Supplementary Figure S3 illustrates the relationship between the partial likelihood deviance (binomial deviance) and the log-transformed penalty parameter (λ) based on 10-fold cross-validation in LASSO regression modeling. Supplementary Figure S4 displays a coefficient profile plot produced against the log (λ) sequence. In this study, optimal values for lambda (λ) were determined at the point of minimum deviance. Alongside λ, the selected mVOCs and covariates for each subgroup analysis are comprehensively detailed for different populations in Supplementary Table S6.

### 3.5 Association of mVOC mixture with RA

**Figure 3:**
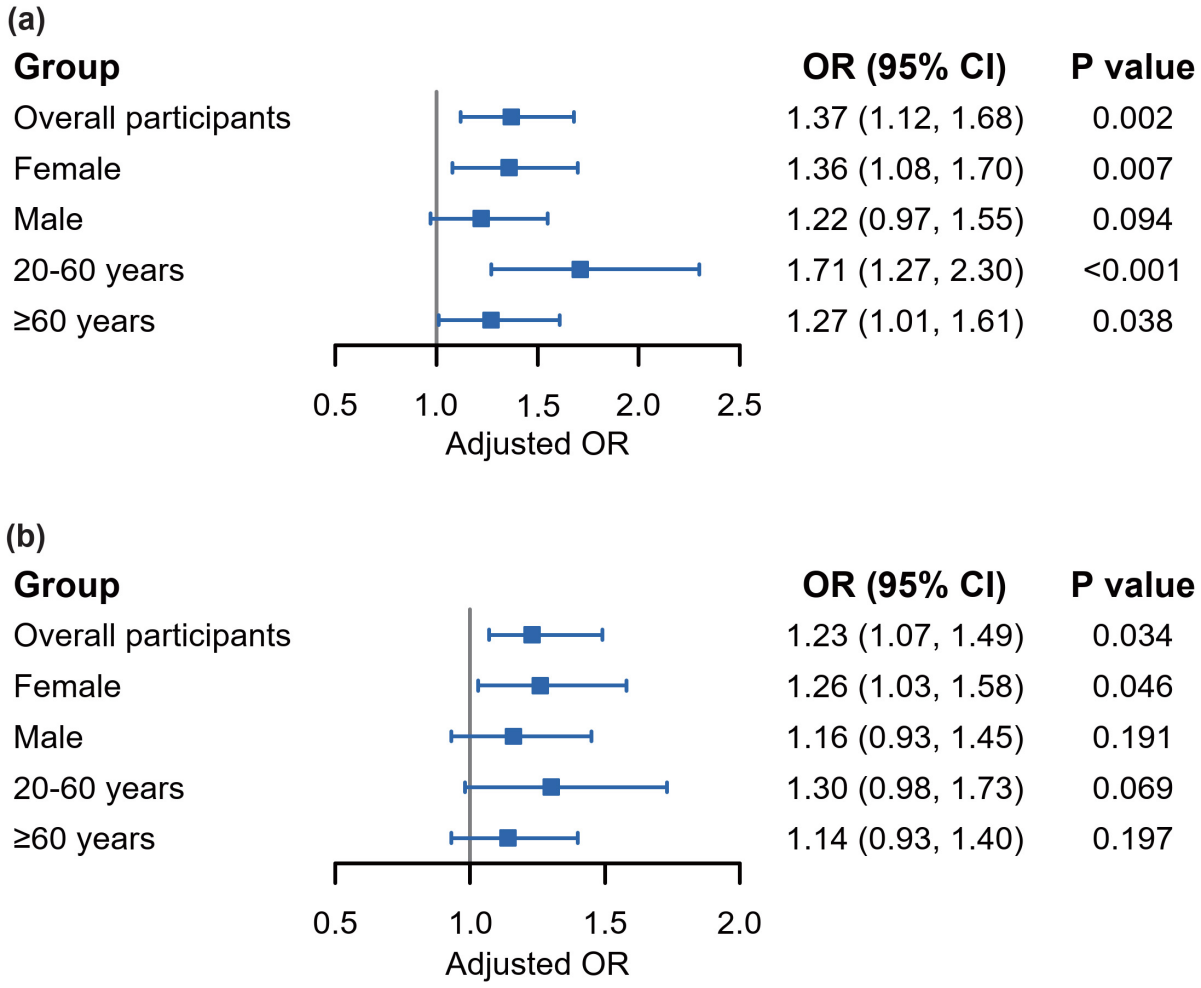
Forest plots demonstrating associations between mVOC mixture and the risk of RA according to WQS regression **(a)** and qgcomp **(b)** analyses. All analyses were adjusted by the covariates selected by LASSO regressions previously conducted. mVOCs, metabolites of volatile organic compounds; RA, rheumatoid arthritis; WQS, weighted quantile sum; qgcomp, quantile g-computation.

The results of WQS regression modeling exploring the combined effect of the multiple-mVOC mixture on RA risk in different subgroups of participants are presented in Figure 3. WQS regression revealed a positive association across the entire cohort of participants (OR: 1.37; 95% CI: 1.12, 1.68; *P* = 0.002), indicating a significantly greater likelihood of RA with increasing mVOC mixture. Stratified analysis indicated that the association remained significant for females (OR: 1.36; 95% CI: 1.08, 1.70; *P* = 0.007) and across all age subgroups within the range of 20-60 years (OR: 1.71; 95% CI: 1.27, 2.30; *P* < 0.001) and ≥60 years (OR: 1.27; 95% CI: 1.01, 1.61; *P* = 0.038). Conversely, the association did not show statistical significance in the male subgroup.

The detailed weights of these mVOCs are presented in Supplementary Figure S5. CYMA emerged as one of the most influential mVOCs for the prevalence of RA, with the highest weights (0.327, 0.822, and 0.395) occurring in the overall cohort population, females, and individuals aged >20–60 years, respectively. This was followed closely by DHBM, CYMA, AMCA, and HPMA in different subpopulations. All these individual variables are significantly associated with RA according to the binary logistic regressions (Supplementary Table S5).

The qgcomp model, which refrains from presupposing a uniform direction for the impact of individual VOC exposures, produced estimated exposure weights that included both positive and negative contributions. The findings are generally consistent with those obtained from the WQS model, suggesting that simultaneous exposure to VOCs is significantly associated with an increased risk of RA across the entire population (OR = 1.23; 95% CI = 1.07, 1.49; *P* = 0.034) and among female participants (OR = 1.26; 95% CI = 1.03, 1.58; *P* = 0.046). Notably, metabolites such as AMCA, DHBM, CYMA, and ATCA were identified as the main contributors to the increase in RA risk in the positive direction. The detailed positive and negative weights attributed to each of the mVOCs and the joint effects are illustrated in Supplementary Figures S6, S7.

### 3.6 Mediation analysis

To further investigate the mechanisms underlying the relationship between mVOCs and the prevalence of RA, mediation analysis was performed with a focus on inflammatory markers. The LMR was used as a representative marker for inflammation. As indicated in Table 3, the LMR played a significant role in mediating the association between mVOCs and RA, with a mediating proportion of 4.65% (95% CI: 4.44, 4.88) (*P* < 0.001) in the overall participants. Stratified analysis showed that the mediating proportions of LMR were 6.52% (*P* < 0.001), 0.77% (*P* < 0.001), 3.39% (*P* < 0.001), and 1.82% (*P* < 0.001) among females, males, individuals aged 20-60 years, and those aged 60 years and above, respectively.

**Table 3:** Mediating effects of inflammatory factors on the association between mVOCs and the risk of RA.

**Subgroup**	**Total effect (95% CI)**	**Mediation effect (95% CI)**	**Direct effect (95% CI)**	**Mediated proportion (%)**	***P*-value**
All participates	0.0051 (0.0050, 0.0053)	0.0002 (0.0002, 0.0003)	0.0049 (0.0048, 0.0050)	4.65 (4.44, 4.88)	<0.001
Female	0.0072 (0.0065, 0.0080)	0.0005 (0.0004, 0.0005)	0.0068 (0.0060, 0.0075)	6.52 (5.87, 7.11)	<0.001
Male	0.0047 (0.0045, 0.0049)	0.0000 (0.0000, 0.0001)	0.0046 (0.0045, 0.0048)	0.77 (0.28, 1.22)	<0.001
20–60 years	0.0039 (0.0037, 0.0041)	0.0284 (0.0230, 0.0340)	0.0037 (0.0036, 0.0039)	3.39 (2.76, 4.02)	<0.001
≥60 years	0.0095 (0.0086, 0.0105)	0.0166 (0.0073, 0.0250)	0.0093 (0.0085, 0.0103)	1.82 (0.79, 2.78)	<0.001

Inflammatory factor levels are represented by the lymphocyte-to-monocyte ratio (LMR). The mediation analyses were adjusted by a comprehensive set of covariates selected by LASSO regression. ACME, average causal mediation effect; ADE, average direct effect.

## 4 Discussion

Our primary objective was to examine associations of individual and multiple co-exposure events to 16 specific VOCs and the prevalence of RA in the U.S. adult population. The key findings of our investigation revealed a clear association, with certain mVOCs (such as AAMA, AMCA, CEMA, CYMA, DHBM, HPMA, MHB3, PHGA, and HPMM) being positively linked to an increased prevalence of RA within the sampled population. Due to the collinearity among different mVOCs, we employed LASSO regression, which is effective at simplifying models by penalizing large coefficients, to identify mVOCs more strongly associated with RA prevalence. Additionally, WQS regression and the qgcomp model showed an elevated risk of RA associated with higher levels of a selected mVOC mixture, with CYMA having the largest contribution to this risk. Finally, mediation analysis revealed that the inflammatory index, as indicated by the LMR, accounted for 4.65% of the mediating effect on the association between multiple VOC co-exposure and RA.

Recent literature has extensively documented the adverse effects of environmental contaminants such as VOCs on human health, linking them to oxidative stress and inflammation in pregnancy, childhood asthma, depression, and cancer (13, 20–23). VOCs are predominantly metabolized in the liver by cytochrome P450 into various hydroxylated and ring-opened compounds and are subsequently excreted in urine (24). Consequently, urinary metabolites can serve as biomarkers for estimating exposure to VOCs (25). To date, the link between mVOC co-exposure and the prevalence of RA has not been thoroughly investigated. A study based on NHANES data revealed a significant association between individual mVOCs, including AMCC and 3HPMA, and the risk of RA (11). However, this study has methodological limitations, chiefly due to the lack of consideration of the statistical collinearity among various mVOCs. Given that mVOCs represent a broad spectrum of substances, often stemming from similar parent compounds, these limitations might skew understanding of their interplay with RA. In addition, it is crucial to consider the typical scenario in which humans are exposed to a mixture of mVOCs rather than to isolated compounds (26, 27). In general, examination of single pollutants in isolation provides limited insight, as it fails to capture potential synergistic or antagonistic interactions that can occur in the pathogenesis of disorders. To address this complexity, our study offers a more realistic and comprehensive assessment of the association between multiple periods of mVOC co-exposure and the risk of RA.

Our results revealed that most individual mVOCs are significantly associated with RA, which is generally consistent with the findings of previous studies (11). More importantly, our findings from multiple-pollutant models also revealed positive correlations between the prevalence of RA and mVOC mixture, with the highest contributors being CYMA, DHBM, AMCA, and ATCA. Stratified analyses further showed that mVOC mixture correlates significantly with RA prevalence within particular subpopulations, specifically females and individuals aged >20–60 years, which are the demographic groups known to have the highest prevalence rates of RA. Furthermore, despite the lack of direct experimental evidence for causality, our mediation analysis for the first time suggests that inflammatory factors may serve as mediators of the relationship between mVOCs and RA. Recognition of inflammatory markers as mediators emphasizes the importance of the inflammatory response in the pathophysiology of RA. This observation is consistent with the prevailing view of RA as an inflammation-driven disease and paves the way for additional investigations into precise preventive approaches. Research into the direct relationships between mVOCs and the risk of RA is relatively scarce. In the field of other musculoskeletal disorders, Zhou et al. reported notable correlations between urinary concentrations of DHBM, AMCA, and ATCA and between bone mineral density and osteoarthritis, suggesting that mVOCs might play a role in altering the bone microenvironment, which may subsequently lead to arthritic inflammation (10, 28, 29).

As mentioned above, inflammation plays a central role in the pathogenesis of RA, and environmental factors, including VOCs, potentially exacerbate these inflammatory pathways and contribute to the disease's onset or progression. Acrylonitrile, which is the precursor to CYMA, is an important monomer in the organic synthesis industry and is widely used in production of synthetic fibers, resins, and plastics (30). Acrylonitrile can be detected in cigarette smoke, followed by drinking water, food, and air, and can be absorbed through ingestion, inhalation of vapors, or dermal contact (31). Research has shown that acrylonitrile can induce an inflammatory response across various cell types, including neuronal cells, testicular cells, oocytes, and gastric mucosal cells (30, 32, 33). This response is characterized by the production of reactive oxygen species (ROS) and the subsequent activation of nuclear factor κB (NF-κB), which are key elements in the cytotoxic effects of the compound observed *in vitro* and eventually lead to synovitis and bone and cartilage degradation (34). DHBM, a metabolite of 1,3-butadiene, has been identified as a significant secondary compound associated with RA. Among the environmental sources of 1,3-butadiene, cigarette smoke stands out as a primary contributor, with other sources including emissions from industrial processes, automobile exhaust, and burning of materials such as wood, plastics, and rubber (35). Numerous studies have investigated the deleterious effects of 1,3-butadiene exposure on diseases involving inflammatory components and the respiratory and cardiovascular systems (36, 37). N,N-dimethylformamide is a parent compound of AMCA, and in addition to generating inflammation similar to the aforementioned effects of acrylonitrile, it can induce neutrophil infiltration and activate the NLRP3 inflammasome in the livers of mice, potentially leading to cellular damage (38). Increased urinary AMCA levels may cause development of inflammatory and fibrotic lesions in the liver through an imbalance in lipid metabolism and the inflammatory response (39). Another study showed that an increased urinary concentration of AMCA impairs lung function through an increased level of C-reactive protein, a commonly used marker of inflammation (40). Cyanide (the precursor of ATCA) is known primarily for its neurotoxic effects (38). Beyond neurotoxicity, cyanide exposure exerts cytotoxicity in non-neuronal cells, as evidenced by an array of damaging cellular events and inflammatory responses (38, 41). These experimental findings suggest a connection between mVOCs and inflammation.

Our study has several strengths. First, it draws upon data that are both nationally representative and encompass a sizable cohort, lending strong credibility to our conclusions. Furthermore, we bolstered the robustness of our analysis by examining the impact of individual VOCs and taking a holistic view of the links between co-exposure to VOCs and RA in overall populations and subpopulations based on sex and age. We utilized a suite of statistical techniques, such as WQS regression, the qgcomp model and mediation analysis, in our assessment, paving the way for a more nuanced grasp of how VOC exposure might influence RA prevalence.

While our study provides valuable insights into the environmental determinants of RA, it is not without limitations. First, self-reported RA diagnosis might introduce reporting bias, though NHANES is known for its rigorous data collection standards. Second, the cross-sectional nature of NHANES data precluded us from establishing a causal relationship between VOC exposure and RA prevalence. This one-time assessment may not accurately reflect chronic exposure, and the possibility of reverse causality, particularly in our mediation analysis, cannot be ruled out. Furthermore, our findings are derived from a U.S. population, and thus may not be generalizable to other populations with different genetic backgrounds, lifestyles, or environmental exposure profiles. Therefore, both longitudinal studies and research in more diverse cohorts are warranted to confirm these associations. Third, although our model incorporated key predictors, we cannot dismiss the possibility that unaccounted factors, such as other environmental air pollutants and covariates, including genetic and occupational factors, might skew the results (42, 43). Fourth, from an initial cohort of 55,810 participants in the selected NHANES cycles, 51,188 were excluded primarily due to missing data on urinary mVOCs and other key covariates, resulting in a final analytical sample of 4,622 participants. This substantial exclusion may have introduced selection bias, potentially limiting the generalizability of our findings to the broader population. Finally, the models (LASSO, WQS and qgcomp) used in this study are not currently adapted for complex survey designs, meaning the necessary NHANES sample weights could not be applied, which may compromise the generalizability of our findings to the broader U.S. population.

## 5 Conclusion

In summary, data from national, cross-sectional studies corroborate the hypothesis that both individual and combined exposures to VOCs are linked to a heightened risk of developing RA. Furthermore, these findings emphasize the significance of inflammatory pathways as potential intermediaries facilitating this connection. Notably, these associations are more pronounced among females and individuals within the young to middle-aged demographic group. Considering the limitations inherent in the present study, prospective cohort studies and experimental research are necessary to verify these associations and clarify the underlying biological mechanisms involved.

## Data Availability

The original contributions presented in the study are included in the article/Supplementary material. The data and analysis code supporting the findings of this study are available at: https://github.com/ruijinyin/Association-between-mVOCs-and-rheumatoid-arthritis. Further inquiries can be directed to the corresponding authors.
