# Supplementary material for: Association between volatile organic compound co-exposure and the prevalence of rheumatoid arthritis: a nationwide cross-sectional study
Source: Front Public Health. 2025 Nov 10;13:1694503. doi: 10.3389/fpubh.2025.1694503 (PMC12640827; doi:10.3389/fpubh.2025.1694503)
Supplement: Supplementary file 1 [file Supplementary_file_1.docx]

# Supplementary materials


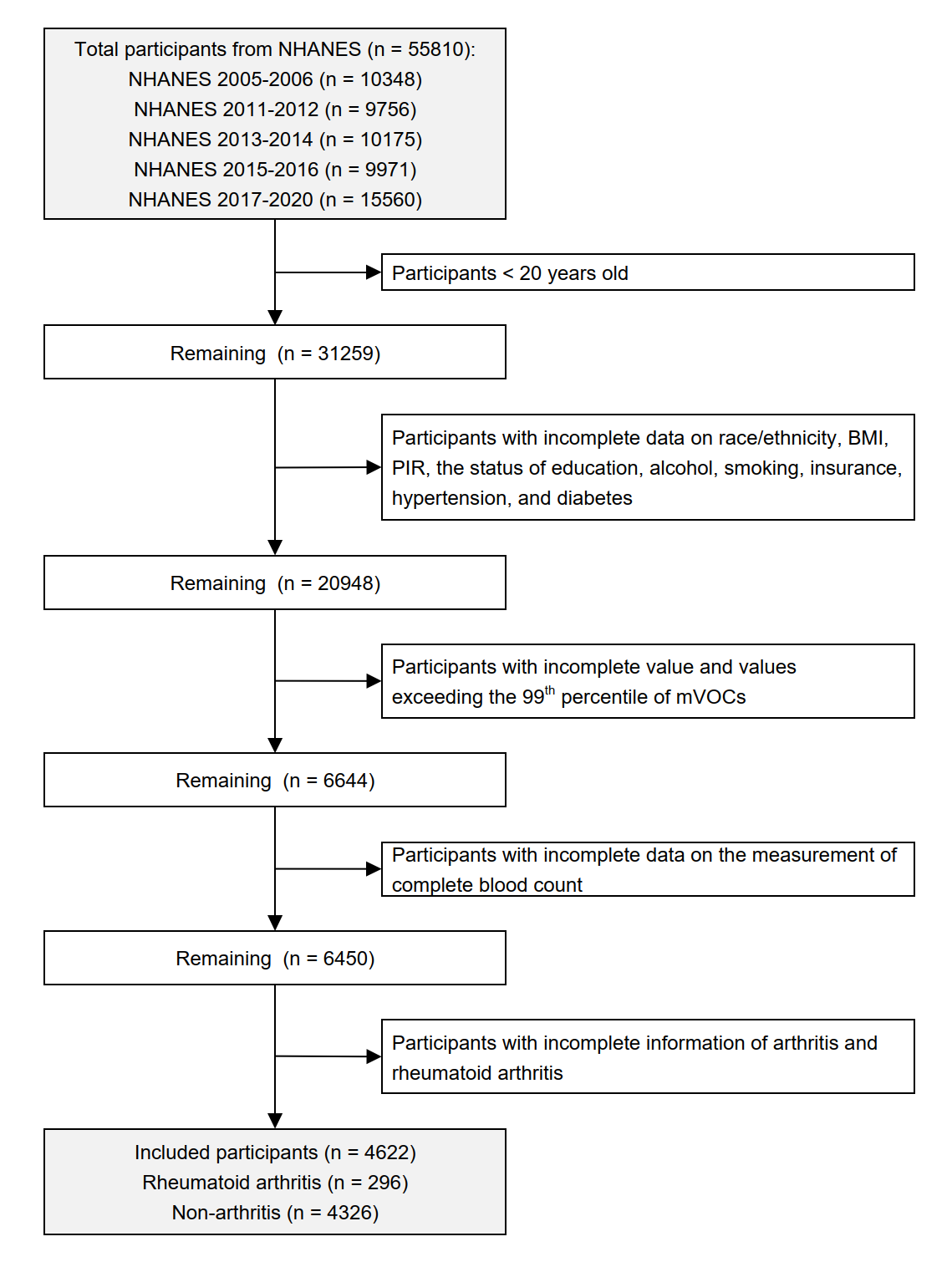


## Fig. S1. The flowchart of the participants selection.

**Abbreviation:** NHANES, National Health and Examination Survey; mVOCs, metabolites of volatile organic compound; BMI, body mass index; PIR, poverty income ratio.

## Table S1. The code, variable name, analyte name, parent compound and LLODs of mVOCs in NHANES 2005-2006, and 2011-2020.

| Code | Variable name | Analyte name (mVOCs) (ng/mL) | Parent compound | LLOD |
| --- | --- | --- | --- | --- |
| AAMA | URXAAM | N-Acetyl-S-(2-carbamoylethyl)-L-cysteine | Acrylamide | 2.2 |
| AMCA | URXAMC | N-Acetyl-S-(N-methylcarbamoyl)-L-cysteine | N, N- Dimethylformamide | 6.26 |
| ATCA | URXATC | 2-Aminothiazoline-4-carboxylic acid | Cyanide | 29.5 |
| BMA | URXBMA | N-Acetyl-S-(benzyl)-L-cysteine | Toluene | 0.5 |
| BPMA | URXBPM | N-Acetyl-S-(n-propyl)-L-cysteine | 1-Bromopropane | 1.2 |
| CEMA | URXCEM | N-Acetyl-S-(2-carboxyethyl)-L-cysteine | Acrolein | 6.96 |
| CYMA | URXCYM | N-Acetyl-S-(2-cyanoethyl)-L-cysteine | Acrylonitrile | 0.5 |
| DHBM | URXDHB | N-Acetyl-S-(3,4-dihydroxybutyl)-L-cysteine | 1,3-Butadiene | 5.25 |
| HPMA | URXHPM | N-Acetyl-S-(3-hydroxypropyl)-L-cysteine | Acrolein | 13 |
| HPM2 | URXHP2 | N-Acetyl-S-(2-hydroxypropyl)-L-cysteine | Propylene oxide | 5.3 |
| MADA | URXMAD | Mandelic acid | Styrene | 12 |
| 2MHA | URX2MH | 2-Methylhippuric acid | Xylene | 5 |
| 34MH | URX34M | 3- and 4-Methylhippuric acid | Xylene | 8 |
| MHB3 | URXMB3 | N-Acetyl-S-(4-hydroxy-2-butenyl)-L-cysteine | 1,3-Butadiene | 0.6 |
| PHGA | URXPHG | Phenylglyoxylic acid | Ethylbenzene, styrene | 12 |
| HPMM | URXPMM | N-Acetyl-S-(3-hydroxypropyl-1-methyl)-L-cysteine | Crotonaldehyde | 1.7 |

**Abbreviation**: mVOCs, metabolites of volatile organic compounds; LLOD, lower limit of detection.

## Table S2. URLs of detailed laboratory measurement of mVOCs and complete blood count from the NHANES 2005-2006, and 2011-2020.

| Laboratory measurement | Stage | URLs |
| --- | --- | --- |
| mVOCs | 2005-2006 | https://wwwn.cdc.gov/Nchs/Nhanes/2005-2006/SSUVOC_D.htm |
|  | 2011-2012 | https://wwwn.cdc.gov/Nchs/Nhanes/2011-2012/UVOC_G.htm |
|  | 2013-2014 | https://wwwn.cdc.gov/Nchs/Nhanes/2013-2014/UVOC_H.htm |
|  | 2015-2016 | https://wwwn.cdc.gov/Nchs/Nhanes/2015-2016/UVOC_I.htm |
|  | 2017-2020 | https://wwwn.cdc.gov/Nchs/Nhanes/2017-2018/P_UVOC.htm |
| Complete blood count | 2005-2006 | https://wwwn.cdc.gov/Nchs/Nhanes/2005-2006/CBC_D.htm |
|  | 2011-2012 | https://wwwn.cdc.gov/Nchs/Nhanes/2011-2012/CBC_G.htm |
|  | 2013-2014 | https://wwwn.cdc.gov/Nchs/Nhanes/2013-2014/CBC_H.htm |
|  | 2015-2016 | https://wwwn.cdc.gov/Nchs/Nhanes/2015-2016/CBC_I.htm |
|  | 2017-2020 | https://wwwn.cdc.gov/Nchs/Nhanes/2017-2018/P_CBC.htm |

**Abbreviation**: mVOCs, volatile organic compounds metabolites.


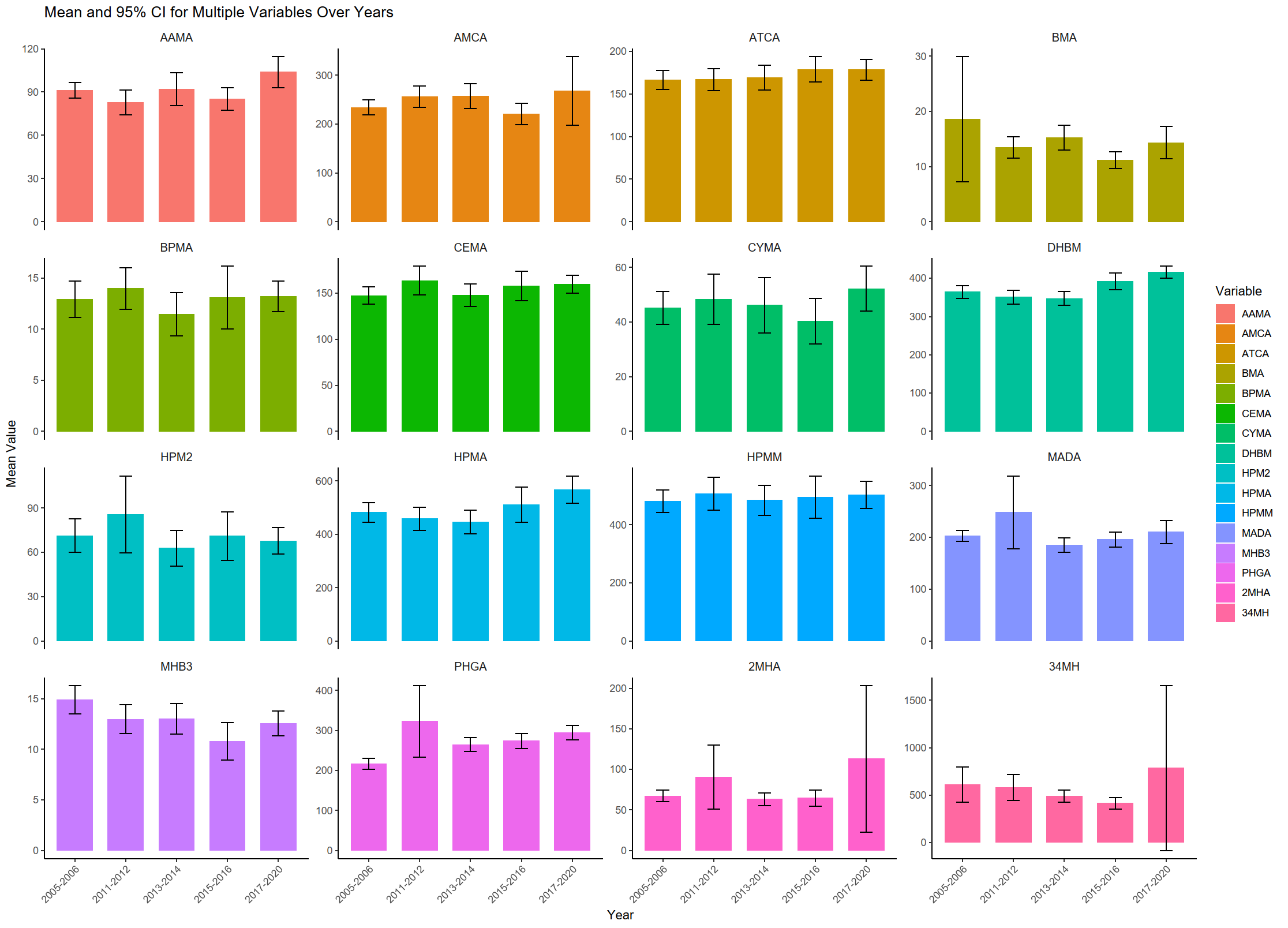


## Fig. S2. Histogram of mVOCs distribution across different NHANES cycles.

**Note**: All values were presented as mean and 95% CI. **Abbreviation**: mVOCs, metabolites of volatile organic compounds.

## Table S3. Percentile distribution and missing value percentage of studied mVOCs.

| mVOCs | 25^th^ percentile | Median | 75^th^ percentile | 95^th^ percentile | 99^th^ percentile | Max value | No. of missing value (%) |
| --- | --- | --- | --- | --- | --- | --- | --- |
| AAMA | 26.8 | 52.3 | 101 | 274 | 584.15 | 5350 | 1306 (7.78) |
| AMCA | 50.4 | 103 | 217 | 696 | 1414.7 | 46300 | 1238 (7.37) |
| ATCA | 56.4 | 137 | 290 | 701 | 1210 | 4480 | 1294 (7.71) |
| BMA | 3.44 | 6.64 | 12.7 | 39.7 | 116.1 | 7930 | 1196 (7.12) |
| BPMA | 0.85 | 3.22 | 9.48 | 42.6 | 114 | 1490 | 1319 (7.85) |
| CEMA | 47.3 | 94.3 | 175 | 452 | 869.34 | 4870 | 1208 (7.19) |
| CYMA | 0.779 | 1.6 | 4.42 | 228 | 570.5 | 2800 | 1241 (7.39) |
| DHBM | 180 | 323 | 517 | 907.55 | 1401.1 | 4580 | 1502 (8.94) |
| HPMA | 122 | 241 | 477 | 1580 | 3732.8 | 27400 | 1405 (8.37) |
| HPM2 | 15.4 | 30.6 | 58.5 | 187 | 612.4 | 10100 | 1221 (7.27) |
| MADA | 72 | 133 | 223 | 485.6 | 991.68 | 75900 | 1283 (7.64) |
| 2MHA | 11.9 | 26 | 62.5 | 211 | 463 | 60300 | 1447 (8.62) |
| 34MH | 74.525 | 162 | 394.75 | 1470 | 3369.8 | 586000 | 1190 (7.09) |
| MHB3 | 2.51 | 4.99 | 10 | 48.86 | 110 | 444 | 1297 (7.72) |
| PHGA | 98.8 | 192 | 328 | 653.65 | 1150 | 74900 | 1244 (7.41) |
| HPMM | 120 | 223 | 400 | 1716.5 | 3980 | 14200 | 1224 (7.29) |

**Abbreviation**: mVOCs, metabolites of volatile organic compounds.

## Table S4. The ln-transformed value of mVOCs according to the status of RA.

| **mVOCs** | **Overall (n = 4622)** | **Non-arthritis (n = 4326)** | **RA (n = 296)** | **P value** |
| --- | --- | --- | --- | --- |
| AAMA | 3.83 (3.19, 4.49) | 3.82 (3.19, 4.47) | 4.02 (3.46, 4.65) | 0.003 |
| AMCA | 4.91 (4.20, 5.63) | 4.88 (4.19, 5.60) | 5.45 (4.60, 6.05) | <0.001 |
| ATCA | 4.54 (3.74, 5.23) | 4.52 (3.74, 5.23) | 4.85 (4.01, 5.40) | 0.014 |
| BMA | 1.79 (1.12, 2.37) | 1.78 (1.10, 2.37) | 1.86 (1.31, 2.29) | 0.295 |
| BPMA | 1.23 (-0.16, 2.23) | 1.23 (-0.16, 2.23) | 1.01 (-0.16, 2.07) | 0.224 |
| CEMA | 4.45 (3.75, 5.05) | 4.42 (3.74, 5.04) | 4.84 (4.37, 5.36) | <0.001 |
| CYMA | 0.41 (-0.30, 1.43) | 0.40 (-0.31, 1.40) | 0.53 (-0.02, 3.36) | 0.008 |
| DHBM | 5.63 (5.02, 6.13) | 5.62 (5.02, 6.12) | 5.89 (5.34, 6.22) | <0.001 |
| HPMA | 5.37 (4.67, 6.02) | 5.35 (4.66, 6.02) | 5.55 (5.10, 6.16) | 0.002 |
| HPM2 | 3.33 (2.67, 3.96) | 3.31 (2.67, 3.95) | 3.46 (2.90, 4.12) | 0.025 |
| MADA | 4.83 (4.23, 5.38) | 4.83 (4.22, 5.37) | 4.93 (4.41, 5.47) | 0.023 |
| 2MHA | 3.31 (2.55, 4.19) | 3.31 (2.55, 4.19) | 3.47 (2.53, 4.27) | 0.622 |
| 34MH | 5.18 (4.34, 6.10) | 5.18 (4.33, 6.09) | 5.20 (4.51, 6.30) | 0.365 |
| MHB3 | 1.48 (0.83, 2.22) | 1.46 (0.82, 2.19) | 1.82 (1.18, 2.62) | 0.001 |
| PHGA | 5.21 (4.56, 5.76) | 5.19 (4.54, 5.76) | 5.40 (4.92, 5.79) | <0.001 |
| HPMM | 5.32 (4.69, 5.95) | 5.31 (4.67, 5.93) | 5.49 (5.14, 6.35) | <0.001 |

**Notes**: All results of mVOCs had been ln-transformed and presented as median (IQR).

## Table S5. Associations of single mVOCs (ln-transformed variable) with the prevalence of RA in overall population included in this study.

| **mVOCs** | **Model 1** | | | |  | **Model 2** | | | |  | **Model 3** | | | |
| --- | --- | --- | --- | --- | --- | --- | --- | --- | --- | --- | --- | --- | --- | --- |
|  | **OR** | **95%CI** | **P value** | **P for trend** |  | **OR** | **95%CI** | **P value** | **P for trend** |  | **OR** | **95%CI** | **P value** | **P for trend** |
| **AAMA** |  |  |  |  |  |  |  |  |  |  |  |  |  |  |
| Q1 | Ref. |  |  | 0.037 |  |  |  |  | 0.003 |  |  |  |  | 0.006 |
| Q2 | 1.56 | 0.74, 3.30 | 0.250 |  |  | 1.57 | 0.74, 3.36 | 0.248 |  |  | 1.50 | 0.70, 3.18 | 0.302 |  |
| Q3 | 2.02 | 1.01, 4.03 | 0.051 |  |  | 2.16 | 1.09, 4.28 | 0.031 |  |  | 2.22 | 1.08, 4.54 | 0.036 |  |
| Q4 | 1.76 | 0.99, 3.11 | 0.057 |  |  | 2.58 | 1.36, 4.90 | 0.005 |  |  | 2.47 | 1.27, 4.79 | 0.011 |  |
| Continuous | 1.18 | 1.02, 1.36 | 0.029 |  |  | 1.36 | 1.13, 1.64 | 0.002 |  |  | 1.34 | 1.10, 1.64 | 0.006 |  |
| **AMCA** |  |  |  |  |  |  |  |  |  |  |  |  |  |  |
| Q1 | Ref. |  |  | 0.002 |  |  |  |  | 0.026 |  |  |  |  | 0.031 |
| Q2 | 0.44 | 0.21, 0.89 | 0.027 |  |  | 0.34 | 0.16, 0.73 | 0.008 |  |  | 0.32 | 0.15, 0.66 | 0.004 |  |
| Q3 | 1.73 | 0.98, 3.08 | 0.065 |  |  | 1.33 | 0.69, 2.57 | 0.398 |  |  | 1.25 | 0.65, 2.43 | 0.507 |  |
| Q4 | 1.76 | 0.93, 3.31 | 0.085 |  |  | 1.43 | 0.72, 2.87 | 0.315 |  |  | 1.42 | 0.72, 2.78 | 0.317 |  |
| Continuous | 1.42 | 1.18, 1.70 | <0.001 |  |  | 1.39 | 1.12, 1.71 | 0.004 |  |  | 1.41 | 1.12, 1.77 | 0.005 |  |
| **ATCA** |  |  |  |  |  |  |  |  |  |  |  |  |  |  |
| Q1 | Ref. |  |  | 0.070 |  |  |  |  | 0.123 |  |  |  |  | 0.159 |
| Q2 | 0.95 | 0.51, 1.77 | 0.863 |  |  | 0.92 | 0.49, 1.73 | 0.800 |  |  | 0.92 | 0.49, 1.72 | 0.796 |  |
| Q3 | 1.63 | 0.85, 3.12 | 0.146 |  |  | 1.41 | 0.74, 2.70 | 0.303 |  |  | 1.44 | 0.71, 2.94 | 0.322 |  |
| Q4 | 1.50 | 0.83, 2.72 | 0.185 |  |  | 1.40 | 0.78, 2.51 | 0.260 |  |  | 1.37 | 0.76, 2.48 | 0.299 |  |
| Continuous | 1.21 | 1.00, 1.46 | 0.050 |  |  | 1.17 | 0.98, 1.40 | 0.088 |  |  | 1.17 | 0.97, 1.41 | 0.110 |  |
| **BMA** |  |  |  |  |  |  |  |  |  |  |  |  |  |  |
| Q1 | Ref. |  |  | 0.300 |  |  |  |  | 0.657 |  |  |  |  | 0.739 |
| Q2 | 2.41 | 1.26, 4.60 | 0.010 |  |  | 2.25 | 1.14, 4.45 | 0.023 |  |  | 2.15 | 1.10, 4.22 | 0.032 |  |
| Q3 | 1.62 | 0.86, 3.07 | 0.142 |  |  | 1.50 | 0.74, 3.02 | 0.262 |  |  | 1.48 | 0.71, 3.08 | 0.298 |  |
| Q4 | 1.54 | 0.90, 2.63 | 0.124 |  |  | 1.31 | 0.73, 2.35 | 0.362 |  |  | 1.25 | 0.71, 2.20 | 0.441 |  |
| Continuous | 1.11 | 0.96, 1.28 | 0.158 |  |  | 1.06 | 0.90, 1.24 | 0.495 |  |  | 1.05 | 0.90, 1.21 | 0.565 |  |
| **BPMA** |  |  |  |  |  |  |  |  |  |  |  |  |  |  |
| Q1 | Ref. |  |  | 0.049 |  |  |  |  | 0.117 |  |  |  |  | 0.232 |
| Q2 | 0.95 | 0.52, 1.73 | 0.866 |  |  | 0.98 | 0.53, 1.82 | 0.943 |  |  | 1.01 | 0.55, 1.88 | 0.964 |  |
| Q3 | 0.85 | 0.46, 1.58 | 0.603 |  |  | 0.93 | 0.48, 1.80 | 0.819 |  |  | 0.91 | 0.47, 1.79 | 0.795 |  |
| Q4 | 0.60 | 0.37, 0.98 | 0.044 |  |  | 0.66 | 0.40, 1.08 | 0.104 |  |  | 0.73 | 0.44, 1.23 | 0.245 |  |
| Continuous | 0.88 | 0.78, 1.01 | 0.065 |  |  | 0.91 | 0.80, 1.04 | 0.165 |  |  | 0.93 | 0.81, 1.06 | 0.297 |  |
| **CEMA** |  |  |  |  |  |  |  |  |  |  |  |  |  |  |
| Q1 | Ref. |  |  | <0.001 |  |  |  |  | <0.001 |  |  |  |  | <0.001 |
| Q2 | 2.49 | 1.33, 4.63 | 0.006 |  |  | 2.23 | 1.14, 4.37 | 0.023 |  |  | 2.44 | 1.22, 4.91 | 0.016 |  |
| Q3 | 3.99 | 2.15, 7.42 | <0.001 |  |  | 3.52 | 1.85, 6.70 | <0.001 |  |  | 3.49 | 1.85, 6.59 | <0.001 |  |
| Q4 | 3.90 | 2.21, 6.89 | <0.001 |  |  | 3.44 | 1.76, 6.73 | 0.001 |  |  | 3.62 | 1.81, 7.26 | 0.001 |  |
| Continuous | 1.49 | 1.29, 1.71 | <0.001 |  |  | 1.39 | 1.15, 1.68 | 0.001 |  |  | 1.41 | 1.15, 1.72 | 0.002 |  |
| **CYMA** |  |  |  |  |  |  |  |  |  |  |  |  |  |  |
| Q1 | Ref. |  |  | 0.221 |  |  |  |  | 0.044 |  |  |  |  | 0.021 |
| Q2 | 1.68 | 0.81, 3.48 | 0.166 |  |  | 1.72 | 0.80, 3.70 | 0.172 |  |  | 1.59 | 0.74, 3.44 | 0.243 |  |
| Q3 | 1.34 | 0.73, 2.46 | 0.345 |  |  | 1.54 | 0.75, 3.16 | 0.240 |  |  | 1.58 | 0.74, 3.35 | 0.243 |  |
| Q4 | 1.62 | 0.86, 3.04 | 0.141 |  |  | 2.29 | 1.12, 4.66 | 0.027 |  |  | 2.23 | 1.19, 4.20 | 0.017 |  |
| Continuous | 1.08 | 1.00, 1.18 | 0.062 |  |  | 1.16 | 1.06, 1.27 | 0.003 |  |  | 1.17 | 1.08, 1.28 | 0.001 |  |
| **DHBM** |  |  |  |  |  |  |  |  |  |  |  |  |  |  |
| Q1 | Ref. |  |  | 0.001 |  |  |  |  | 0.004 |  |  |  |  | 0.013 |
| Q2 | 1.42 | 0.74, 2.73 | 0.299 |  |  | 1.18 | 0.60, 2.30 | 0.635 |  |  | 1.19 | 0.58, 2.44 | 0.629 |  |
| Q3 | 2.60 | 1.34, 5.05 | 0.006 |  |  | 2.37 | 1.13, 4.95 | 0.026 |  |  | 2.20 | 1.07, 4.52 | 0.039 |  |
| Q4 | 2.25 | 1.32, 3.82 | 0.004 |  |  | 2.18 | 1.21, 3.93 | 0.012 |  |  | 2.00 | 1.06, 3.77 | 0.037 |  |
| Continuous | 1.46 | 1.20, 1.79 | <0.001 |  |  | 1.42 | 1.11, 1.81 | 0.008 |  |  | 1.35 | 1.04, 1.75 | 0.030 |  |
| **HPMA** |  |  |  |  |  |  |  |  |  |  |  |  |  |  |
| Q1 | Ref. |  |  | 0.065 |  |  |  |  | 0.040 |  |  |  |  | 0.025 |
| Q2 | 2.36 | 1.28, 4.36 | 0.008 |  |  | 2.59 | 1.28, 5.25 | 0.011 |  |  | 2.55 | 1.25, 5.23 | 0.014 |  |
| Q3 | 1.82 | 1.07, 3.08 | 0.030 |  |  | 2.24 | 1.17, 4.27 | 0.018 |  |  | 2.22 | 1.16, 4.24 | 0.021 |  |
| Q4 | 1.92 | 1.09, 3.40 | 0.029 |  |  | 2.37 | 1.15, 4.89 | 0.024 |  |  | 2.52 | 1.21, 5.25 | 0.018 |  |
| Continuous | 1.18 | 1.01, 1.37 | 0.040 |  |  | 1.28 | 1.06, 1.54 | 0.015 |  |  | 1.30 | 1.08, 1.58 | 0.009 |  |
| **HPM2** |  |  |  |  |  |  |  |  |  |  |  |  |  |  |
| Q1 | Ref. |  |  | 0.319 |  |  |  |  | 0.178 |  |  |  |  | 0.179 |
| Q2 | 1.43 | 0.71, 2.91 | 0.321 |  |  | 1.65 | 0.80, 3.40 | 0.182 |  |  | 1.53 | 0.79, 2.96 | 0.220 |  |
| Q3 | 1.30 | 0.69, 2.44 | 0.423 |  |  | 1.53 | 0.79, 2.97 | 0.215 |  |  | 1.51 | 0.76, 3.00 | 0.242 |  |
| Q4 | 1.37 | 0.81, 2.30 | 0.243 |  |  | 1.56 | 0.90, 2.70 | 0.119 |  |  | 1.52 | 0.89, 2.59 | 0.135 |  |
| Continuous | 1.08 | 0.96, 1.22 | 0.210 |  |  | 1.11 | 0.98, 1.26 | 0.119 |  |  | 1.10 | 0.96, 1.27 | 0.185 |  |
| **MADA** |  |  |  |  |  |  |  |  |  |  |  |  |  |  |
| Q1 | Ref. |  |  | 0.125 |  |  |  |  | 0.059 |  |  |  |  | 0.122 |
| Q2 | 1.94 | 1.03, 3.67 | 0.045 |  |  | 1.89 | 0.96, 3.71 | 0.070 |  |  | 1.80 | 0.90, 3.61 | 0.104 |  |
| Q3 | 1.68 | 0.89, 3.19 | 0.117 |  |  | 1.78 | 0.84, 3.79 | 0.141 |  |  | 1.71 | 0.81, 3.61 | 0.170 |  |
| Q4 | 1.60 | 0.97, 2.63 | 0.069 |  |  | 1.93 | 1.09, 3.44 | 0.029 |  |  | 1.82 | 0.95, 3.50 | 0.078 |  |
| Continuous | 1.15 | 0.98, 1.36 | 0.097 |  |  | 1.24 | 0.99, 1.54 | 0.061 |  |  | 1.21 | 0.97, 1.51 | 0.103 |  |
| **2MHA** |  |  |  |  |  |  |  |  |  |  |  |  |  |  |
| Q1 | Ref. |  |  | 0.840 |  |  |  |  | 0.478 |  |  |  |  | 0.352 |
| Q2 | 0.87 | 0.47, 1.63 | 0.668 |  |  | 0.86 | 0.45, 1.65 | 0.661 |  |  | 0.90 | 0.48, 1.70 | 0.758 |  |
| Q3 | 1.24 | 0.69, 2.24 | 0.472 |  |  | 1.31 | 0.70, 2.46 | 0.395 |  |  | 1.42 | 0.72, 2.80 | 0.311 |  |
| Q4 | 0.95 | 0.51, 1.78 | 0.871 |  |  | 1.11 | 0.57, 2.18 | 0.755 |  |  | 1.20 | 0.62, 2.29 | 0.592 |  |
| Continuous | 0.97 | 0.82, 1.14 | 0.673 |  |  | 1.02 | 0.85, 1.21 | 0.853 |  |  | 1.03 | 0.87, 1.22 | 0.756 |  |
| **34MH** |  |  |  |  |  |  |  |  |  |  |  |  |  |  |
| Q1 | Ref. |  |  | 0.792 |  |  |  |  | 0.861 |  |  |  |  | 0.955 |
| Q2 | 1.03 | 0.54, 1.94 | 0.932 |  |  | 0.88 | 0.44, 1.74 | 0.711 |  |  | 0.84 | 0.42, 1.68 | 0.619 |  |
| Q3 | 0.77 | 0.42, 1.43 | 0.420 |  |  | 0.68 | 0.37, 1.27 | 0.234 |  |  | 0.70 | 0.37, 1.34 | 0.289 |  |
| Q4 | 1.01 | 0.56, 1.83 | 0.966 |  |  | 1.03 | 0.54, 1.96 | 0.939 |  |  | 1.04 | 0.57, 1.90 | 0.894 |  |
| Continuous | 1.00 | 0.87, 1.15 | 0.998 |  |  | 0.99 | 0.85, 1.16 | 0.935 |  |  | 0.99 | 0.85, 1.15 | 0.878 |  |
| **MHB3** |  |  |  |  |  |  |  |  |  |  |  |  |  |  |
| Q1 | Ref. |  |  | 0.008 |  |  |  |  | 0.008 |  |  |  |  | 0.009 |
| Q2 | 1.86 | 0.81, 4.29 | 0.150 |  |  | 1.71 | 0.72, 4.06 | 0.226 |  |  | 1.59 | 0.68, 3.71 | 0.291 |  |
| Q3 | 2.12 | 1.07, 4.21 | 0.036 |  |  | 2.13 | 1.00, 4.55 | 0.057 |  |  | 2.15 | 0.99, 4.66 | 0.059 |  |
| Q4 | 2.39 | 1.26, 4.53 | 0.010 |  |  | 2.67 | 1.29, 5.52 | 0.010 |  |  | 2.61 | 1.29, 5.29 | 0.011 |  |
| Continuous | 1.22 | 1.05, 1.43 | 0.013 |  |  | 1.28 | 1.06, 1.54 | 0.013 |  |  | 1.29 | 1.05, 1.57 | 0.018 |  |
| **PHGA** |  |  |  |  |  |  |  |  |  |  |  |  |  |  |
| Q1 | Ref. |  |  | <0.001 |  |  |  |  | 0.002 |  |  |  |  | 0.007 |
| Q2 | 1.97 | 1.10, 3.55 | 0.027 |  |  | 1.62 | 0.91, 2.88 | 0.109 |  |  | 1.76 | 0.95, 3.28 | 0.080 |  |
| Q3 | 3.70 | 2.12, 6.47 | <0.001 |  |  | 2.98 | 1.66, 5.33 | 0.001 |  |  | 2.87 | 1.53, 5.38 | 0.002 |  |
| Q4 | 2.16 | 1.31, 3.55 | 0.004 |  |  | 2.07 | 1.23, 3.49 | 0.009 |  |  | 2.04 | 1.15, 3.60 | 0.019 |  |
| Continuous | 1.22 | 1.08, 1.38 | 0.002 |  |  | 1.21 | 1.04, 1.41 | 0.017 |  |  | 1.17 | 1.00, 1.37 | 0.062 |  |
| **HPMM** |  |  |  |  |  |  |  |  |  |  |  |  |  |  |
| Q1 | Ref. |  |  | 0.007 |  |  |  |  | 0.017 |  |  |  |  | 0.014 |
| Q2 | 1.80 | 0.80, 4.06 | 0.160 |  |  | 1.61 | 0.70, 3.71 | 0.271 |  |  | 1.50 | 0.63, 3.56 | 0.369 |  |
| Q3 | 1.49 | 0.79, 2.83 | 0.226 |  |  | 1.43 | 0.69, 2.97 | 0.344 |  |  | 1.28 | 0.61, 2.68 | 0.510 |  |
| Q4 | 2.43 | 1.33, 4.45 | 0.006 |  |  | 2.44 | 1.25, 4.78 | 0.012 |  |  | 2.38 | 1.27, 4.49 | 0.010 |  |
| Continuous | 1.28 | 1.09, 1.50 | 0.003 |  |  | 1.31 | 1.09, 1.58 | 0.007 |  |  | 1.30 | 1.08, 1.55 | 0.007 |  |

**Notes**: All results of mVOCs had been ln-transformed, and the first, second, third, and fourth quartiles of concentration were denoted by Q1 (P_0–25_), Q2 (P_25–50_), Q3 (P_50–75_) and Q4 (P_75–100_). The quartile values and continuous values of mVOCs were both subjected to logistic regression analysis to assess their association with RA risk. Model 1: non-adjusted. Model 2: adjusted by age, sex, race, BMI levels, and waist circumference. Model 3: further adjusted by education level, health insurance, poverty income ratio, smoking status, alcohol intake, hypertension, and diabetes status based on Model 2. **Abbreviation**: RA, rheumatoid arthritis; mVOCs, metabolites of volatile organic compounds; OR, odds ratio; CI, confidence interval.


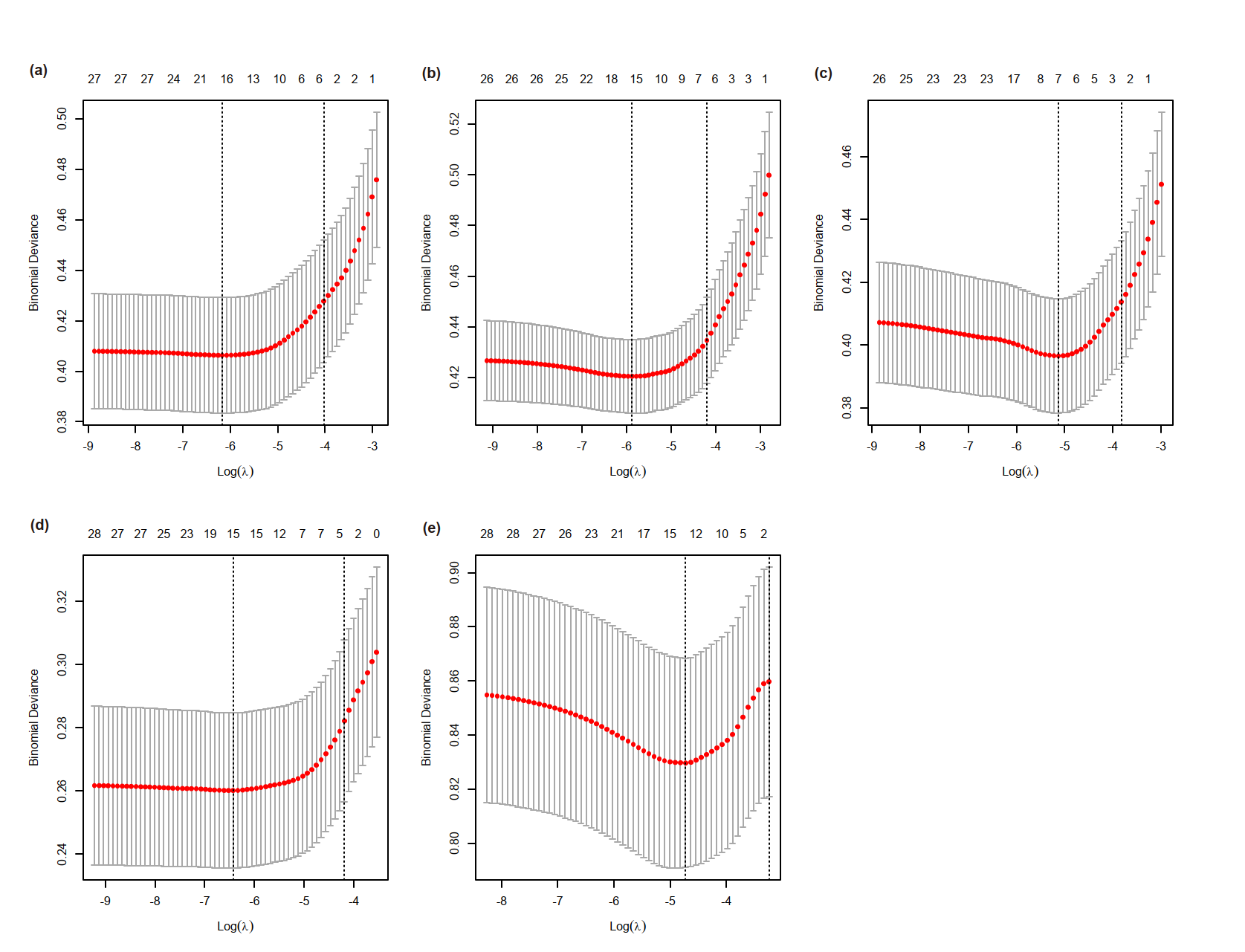


## Fig. S3. Association of the variation of binomial deviance with the log-transformed penalty parameter (log λ) in LASSO regression modeling.

Note: The model included standardized ln-transformed urinary levels of 16 creatinine-corrected mVOCs as independent variables. Covariates encompassed age, sex, race, BMI levels, and waist circumference, education, health insurance, poverty income ratio, smoking status, alcohol intake, hypertension, and diabetes status. The red dot signifies the average binomial deviance, while the solid lines flanking it represent the 95% CIs. Dotted vertical lines have been drawn at the points representing the optimal values derived from the minimum criteria and at the value one standard error above the minimum criteria. The dependent variables were the prevalence of RA at various sub-populations: the entire cohort of participants (a), females (b), males (c), individuals aged 20-60 years (d), and those aged 60 years and above (e).


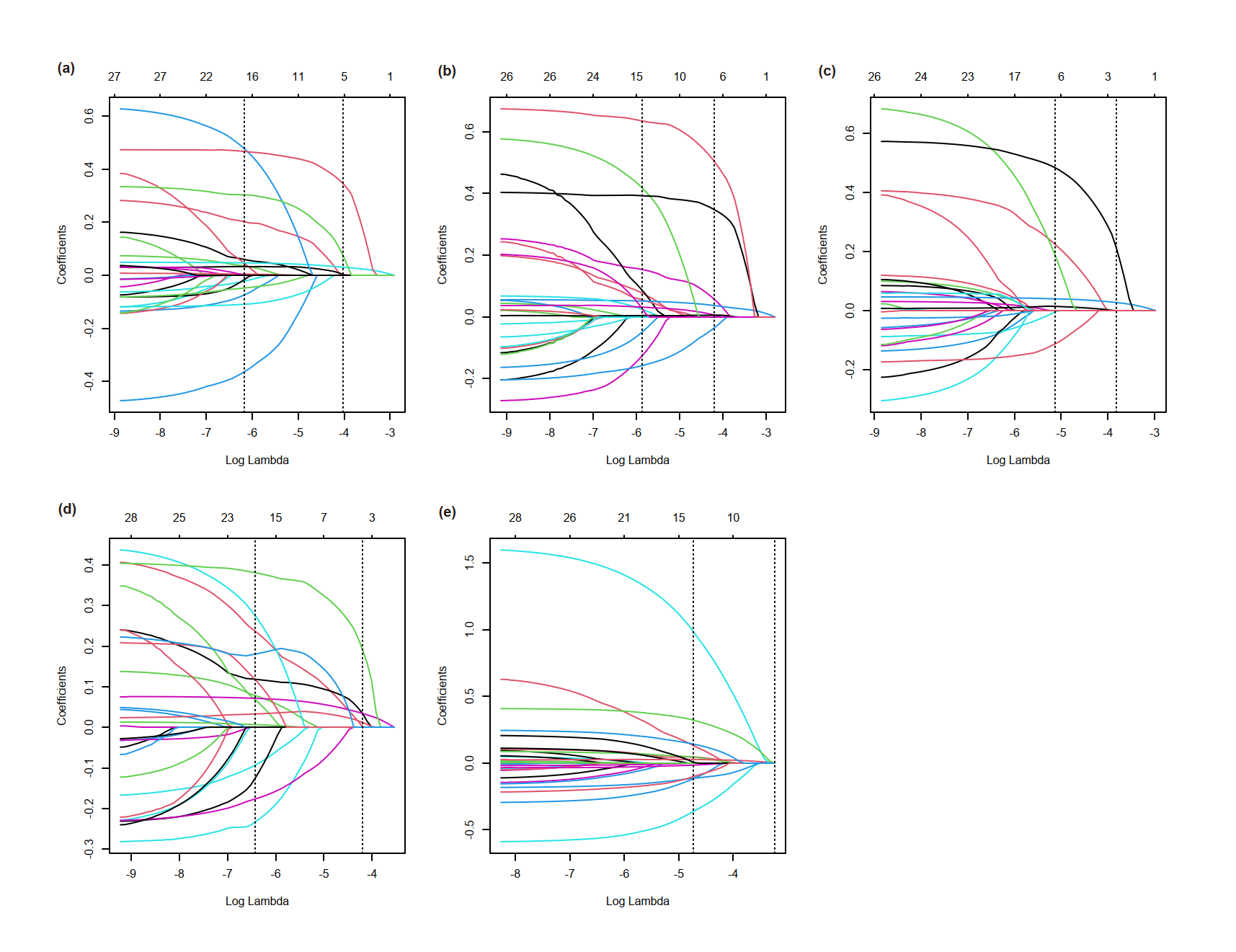


## Fig. S4. The β shrinkage process in the LASSO regression for identifying mVOCs and covariates most significantly associated with RA.

Note: The regression model employed independent variables consisting of ln-transformed urinary levels of 16 creatinine-corrected mVOCs. A range of covariates was integrated into the model, including age, sex, race, BMI, waist circumference, education level, health insurance status, poverty income ratio, smoking habits, alcohol consumption, hypertension, and diabetes status. The dependent variables targeted by the regression analysis were the risk of RA across various sub-populations: the entire cohort of participants (a), females (b), males (c), individuals aged 20-60 years (d), and those aged 60 years and above (e).

## Table S6. The selected variables and covariates according to LASSO regression in different group for the further analysis.

| **Group** | **Optimal λ** | **Log(λ)** | **Selected mVOCs variables and covariates** |
| --- | --- | --- | --- |
| All participants | 0.00209341 | -6.168961 | mVOCs: AMCA, ATCA, BMA, BPMA, CYMA, DHBM, 34MH  Covariates: sex, age, BMI, waist circumference, education level, health insurance, poverty income ratio, smoking habits, hypertension, diabetes |
| Female | 0.002809108 | -5.874888 | mVOCs: AMCA, BMA, CYMA, DHBM, HPMA, 34MH  Covariates: age, race/ethnicity, BMI, waist circumference, health insurance, poverty income ratio, alcohol intake, hypertension, diabetes |
| Male | 0.005934251 | -5.127014 | mVOCs: AMCA, BPMA  Covariates: age, waist circumference, education level, health insurance, hypertension |
| 20-60 years | 0.001616054 | -6.427768 | mVOCs: AMCA, ATCA, BPMA, CYMA, HPMA, 34MH  Covariates: sex, age, BMI, waist circumference, health insurance, poverty income ratio, alcohol intake, hypertension, diabetes |
| ≥60 years | 0.008844959 | -4.727908 | mVOCs: AMCA, BMA, DHBM, PHGA  Covariates: sex, age, BMI, education level, health insurance, poverty income ratio, smoking habits, alcohol intake, hypertension, diabetes |

Note: Optimal λ was set at the minimum criteria of binomial deviance for all sub-population. Abbreviation: mVOCs, metabolites of volatile organic compounds; BMI, body mass index; PIR, poverty income ratio.


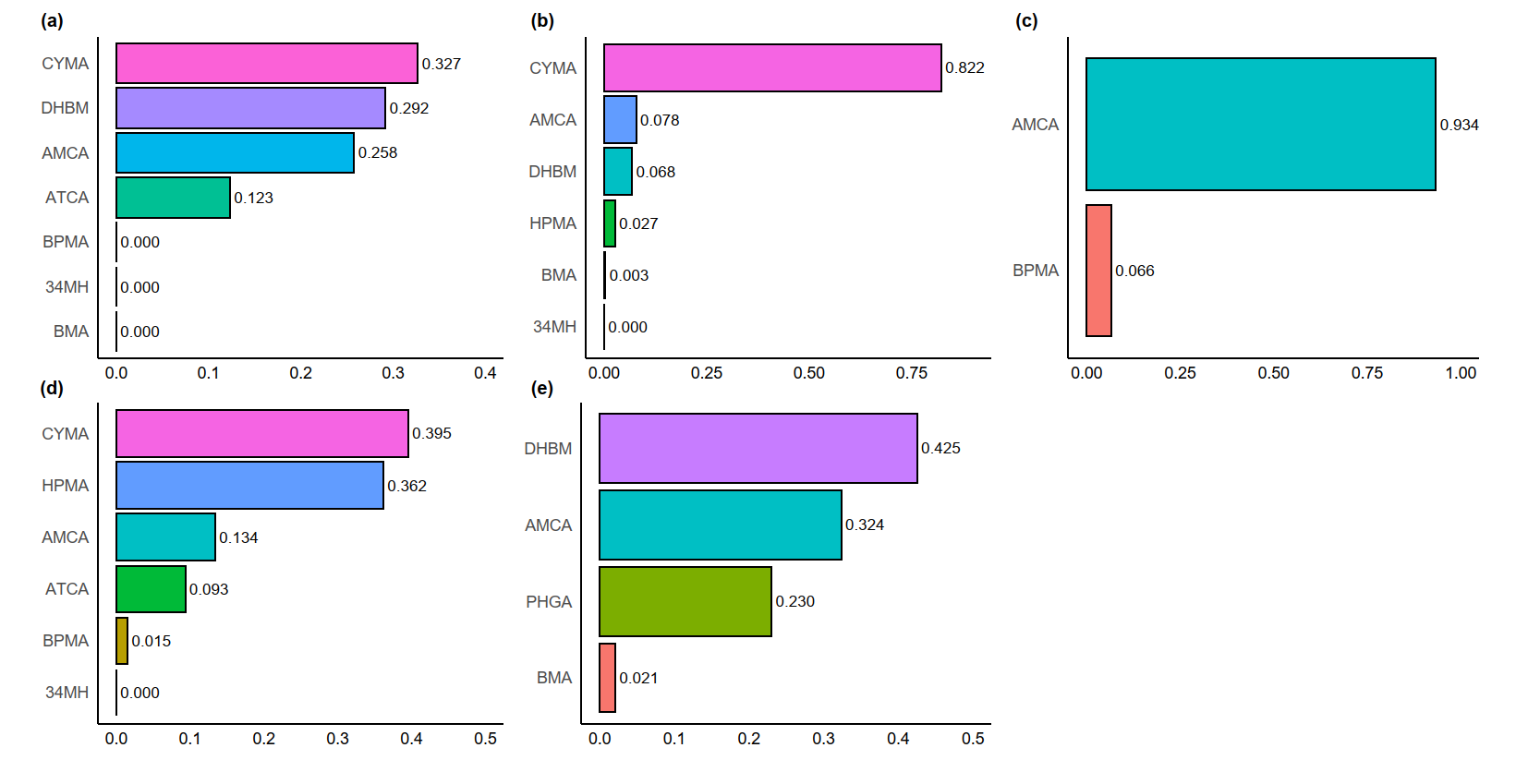


## Fig. S5. Estimated weights of urinary mVOCs for the prevalence of RA by WQS models in the entire cohort of participants (a) and 4 sub-populations including females (b), males (c), individuals aged 20-60 years (d), and those aged 60 years and above (e).


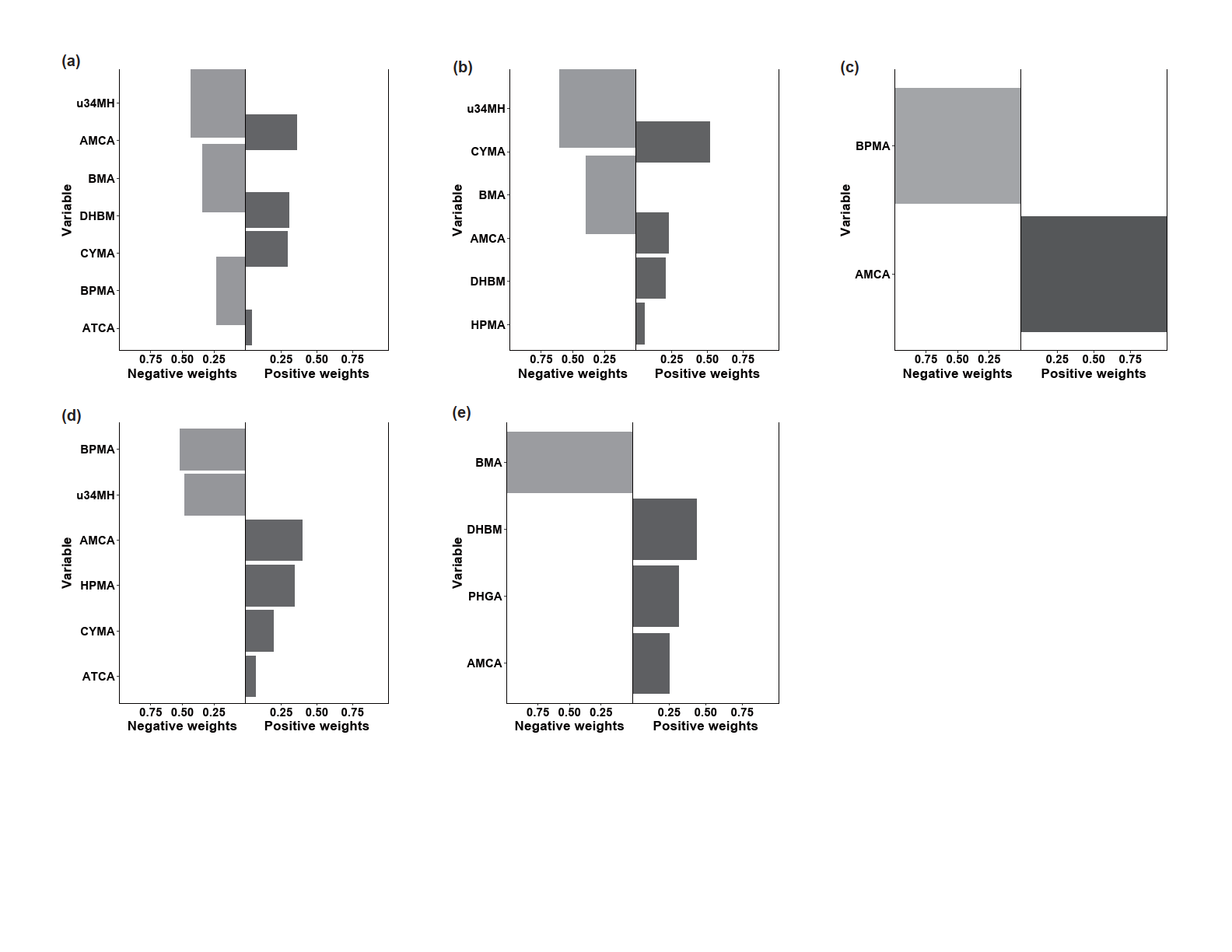


## Fig. S6. The weights from the qgcomp model regression for urinary mVOCs in relation to the prevalence of RA across the entire cohort of participants (a) and within four sub-populations, namely females (b), males (c), individuals aged 20-60 years (d), and those aged 60 years and above (e).

Note: The central vertical line indicates a weight of zero. Values to the right suggest a positive correlation between mVOCs and RA risk, whereas values to the left indicate a negative correlation.


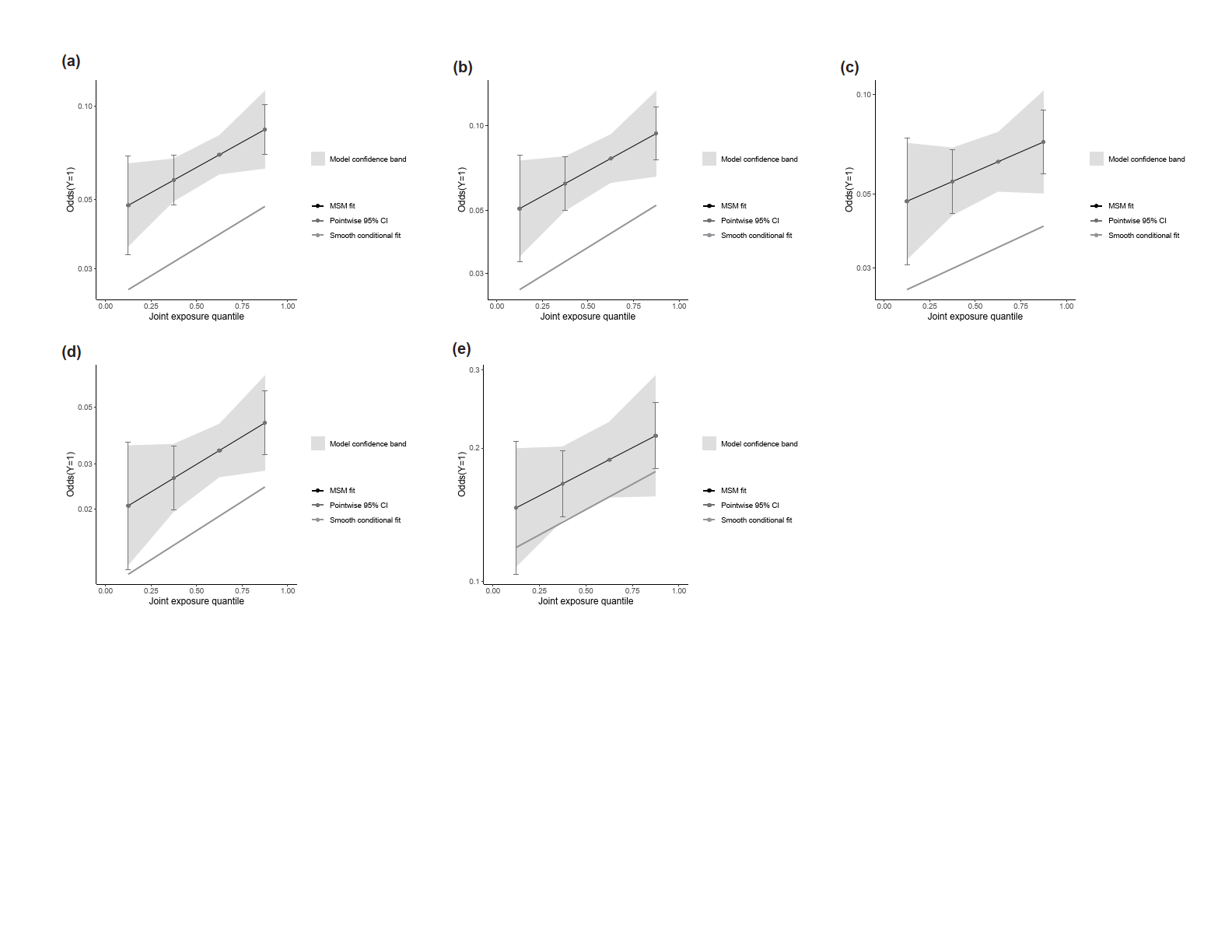


## Fig. S7. The joint effect (95% CI) in qgcomp model for urinary mVOCs on the prevalence of RA in the entire cohort of participants (a), as well as within four sub-groups including females (b), males (c), individuals aged 20-60 years (d), and those aged 60 years and above (e).
